# Blastomatoid pulmonary carcinosarcoma: report of a case with a review of the literature

**DOI:** 10.1186/1471-2407-12-424

**Published:** 2012-09-25

**Authors:** Inga-Marie Schaefer, Carsten-Oliver Sahlmann, Tobias Overbeck, Stefan Schweyer, Jan Menke

**Affiliations:** 1 Department of Pathology, University Medical Center Göttingen, Robert-Koch-Straße 40, D-37075, Göttingen, Germany; 2 Nuclear Medicine, University Medical Center Göttingen, Göttingen, Germany; 3 Haematology and Oncology, University Medical Center Göttingen, Göttingen, Germany; 4 Diagnostic Radiology, University Medical Center Göttingen, Göttingen, Germany

**Keywords:** Lung, biphasic, Carcinosarcoma, Pulmonary blastoma, Comparative genomic hybridization

## Abstract

**Background:**

Pulmonary carcinosarcoma is a biphasic tumour with an unfavourable prognosis. The differential diagnosis includes pulmonary blastoma and is often challenging.

**Case presentation:**

We here describe a case of blastomatoid pulmonary carcinosarcoma in a 58-year-old patient, who underwent surgical resection. Histopathological examination revealed immature glandular epithelium resembling high-grade fetal adenocarcinoma expressing epithelial markers and membranous beta-catenin, and blastomatoid spindle cells with partial rhabdomyosarcoma-like differentiation. Both elements expressed p53, MDM2, and cyclin-dependent kinase 4 (CDK4), but not thyroid-transcription factor 1 (TTF-1). Mutation analysis of *KRAS*, *EGFR*, and *beta-catenin* revealed no mutations. Comparative genomic hybridization detected +1q, +6p, +6q24qter, +8q, +11q12q14, +11q23qter, +12q12q21, +12q24qter, +17q, +20q, -5q14q23, -9p13pter, -13q21q21, and amplifications at 12q14q21, 15q24qter, 20q11q12.

**Conclusion:**

The observed molecular and cytogenetic findings may provide additional tools for the differential diagnosis of biphasic pulmonary neoplasms. Furthermore, *TP53*, *MDM2*, *CDK4*, and *PTPN1* may be involved in tumourigenesis.

## Background

The differential diagnosis of biphasic pulmonary tumours consisting of a mesenchymal component combined with adenocarcinoma includes pulmonary blastoma and carcinosarcoma [1-3]. The degree and type of differentiation of both elements, immunohistochemical staining and molecular genetic findings are of help in establishing the correct diagnosis, which may be challenging in some cases. However, the developmental origin and the correct classification of pulmonary blastoma and carcinosarcoma is still being discussed on [1]. Here, we describe a case of pulmonary carcinosarcoma of the blastomatoid variant in a 58-year-old patient who underwent pneumonectomy. We present the clinico-pathological characteristics and the results of immunohistochemistry, mutation analysis, chromogenic in situ hybridization (CISH), and comparative genomic hybridization (CGH) of this rare entity with a review of the literature, providing helpful diagnostic tools in the differential diagnosis.

## Case presentation

**Figure 1:**
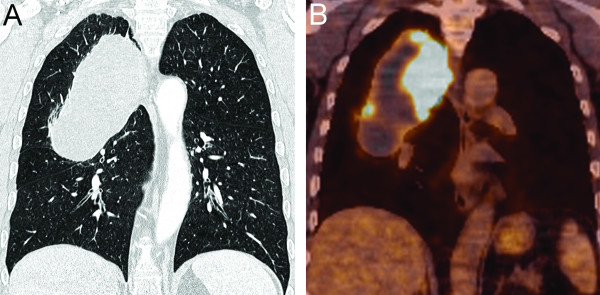
**Radiographic findings of the blastomatoid pulmonary carcinosarcoma.** Preoperative computed tomography (CT) detected a sharply marginated tumour in the upper right hemithorax of up to 12.9 cm size (**A**, coronal view). The tumour extended to the right hilus, pleura, and the spinal column. Positron emission tomography with integrated computed tomography (FDG-PET/CT, Philips Gemini, PET-acquisition 90 minutes after intravenous injection of 198 megabecquerel 2-(^18^F)-fluoro-2-deoxy-D-glucose) revealed marked FDG-glucose uptake in the pulmonary mass, especially median, lateral and caudal, with a maximum standardized uptake value (SUV_max_) of 14.1 (**B**, fusion PET/CT, coronal view).

**Figure 2:**
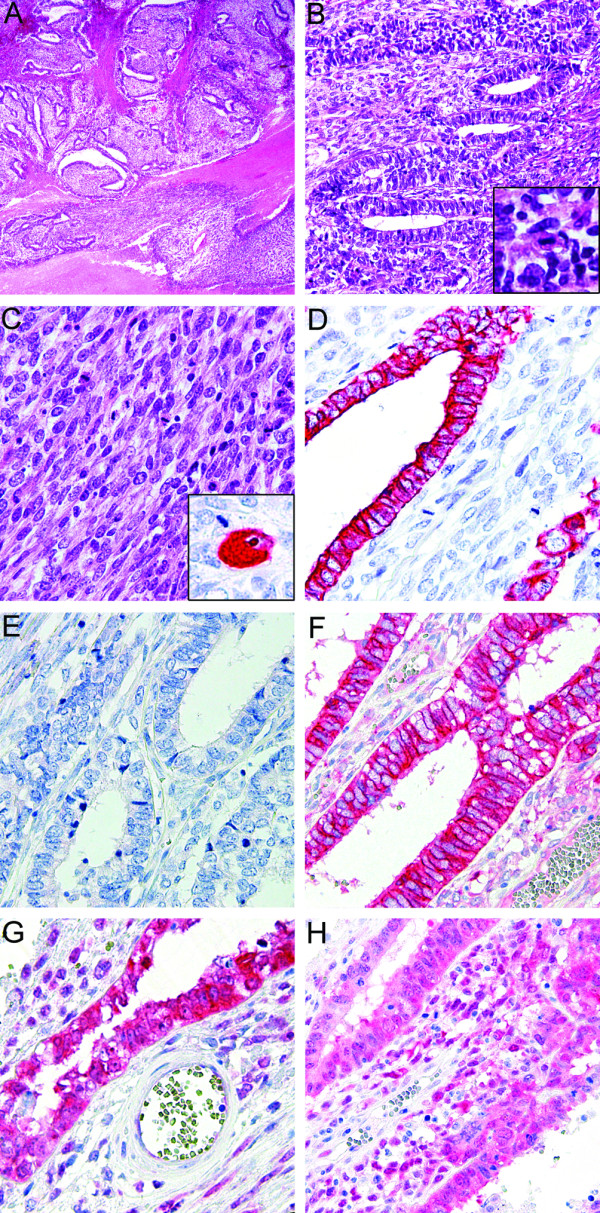
**Microscopic findings of the blastomatoid pulmonary carcinosarcoma.** On microscopic view, the blastomatoid pulmonary carcinosarcoma displayed a biphasic growth pattern (**A**, haematoxylin-eosin (HE), x 20). The epithelial elements showed glandular differentiation (**B**, x 100) and displayed focal cellular atypia and mitotic figures (**B**, inset) resembling high-grade fetal adenocarcinoma. These structures were surrounded by malignant, blastomatoid spindle cells (**C**, x 200), which showed partial rhabdomyosarcomatous differentiation with rhabdomyoblasts (**C**, inset, desmin). Immunohistochemical staining with pan-cytokeratin (**D**) was positive in the carcinomatous areas. Both components did not express thyroid transcription factor 1 (TTF-1; **E**). Membranous beta-catenin (**F**) expression was detected in the epithelial structures; MDM2 (**G**) and cyclin-dependent kinase 4 (CDK4; **H**) were expressed in both components (x 200).

A 58-year-old male patient was admitted to hospital after suffering for several weeks from shoulder pain, dyspnoea, and cough. His medical history included chronic obstructive pulmonary disease and smoking (80 pack years). Computed tomography (CT) detected a 12.9-cm sharply marginated mass lesion in the upper right hemithorax with central necroses and marked FDG-glucose uptake in positron emission tomography with integrated computed tomography (Figure 1). There was no evidence of distant metastases. Histopathological examination of a CT-guided biopsy revealed malignant tumour cells with epithelial and mesenchymal differentiation. Right-sided pneumectomy with lymphadenectomy was performed, rendering a well circumscribed tumour of 15 x 9 x 6 cm size with a soft fleshy, tan-white, lobulated cut surface. Chest wall or pleura were not infiltrated. Microscopically, the lesion was composed of areas of glandular differentiation and immature spindle cell areas with a sharp border between both elements (Figure 2). The tumour exhibited numerous atypical mitotic figures, rich vascularization, and regressive changes including calcifications and extensive necroses. The epithelial component, accounting for ~40% of the vital tumour, showed primitive differentiation, in some areas with nuclear atypia and a branching (immature) architecture of glands composed of columnar cells with palisading elliptic nuclei with subnuclear vacuoles suggestive of high-grade fetal adenocarcinoma.

Immunohistochemically, the glandular structures expressed epithelial markers including epithelial membrane antigen (EMA; Dako, Glostrup, Denmark), pan-cytokeratin (Dako), carcinoembryonic antigen (CEA; Zytomed Systems, Berlin, Germany), CK7 (Dako), and focal p63 (BioGenex, San Ramon, CA). Thyroid transcription factor 1 (TTF-1; Dako) was not expressed. Staining for beta-catenin (Medac, Wedel, Germany) demonstrated a membranous expression in the epithelial structures. The spindle cell component expressed vimentin (Dako), desmin (Invitrogen, Camarillo, CA) in 10% of tumour cells, and myogenin (Dako) in 10% of tumour cells indicating partial rhabdomyosarcoma-like differentiation, EGFR (Invitrogen), and CD56 (Invitrogen). Both components expressed p53 (Dako), MDM2 (Zytomed Systems), and cyclin-dependent kinase 4 (CDK4; Zytomed Systems). Proliferative activity was assessed by Ki67 (Zytomed Systems) immunostaining and was estimated at 60%.

**Figure 3:**
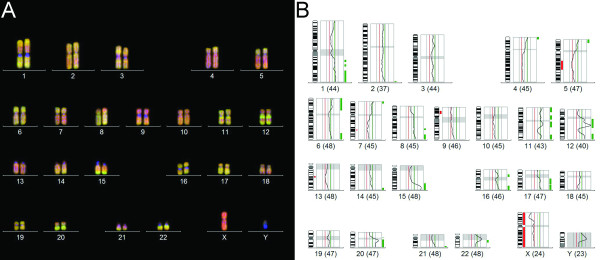
**Results of comparative genomic hybridization (CGH).** CGH of the blastomatoid pulmonary carcinosarcoma revealed ish cgh enh(1)(q),dim(5)(q14q23),enh (6)(p),enh(6)(q24qter),enh(8)(q),dim(9)(p13pter),enh(11)(q12q14),enh(11)(q23qter),enh(12)(q12q21),amp(12)(q14q21),enh(12)(q24qter),dim(13)(q21q21),amp(15)(q24qter),enh(17)(q),enh(20)(q),amp(20)(q11q12). The number of chromosomes included in the CGH analysis is indicated at the bottom of each individual profile.

A *SYT-SSX* fusion gene suggestive of synovial sarcoma was not detected by RT-PCR. Sequencing analysis of *KRAS* exon 1 and 2, *EGFR* exon 19, 20, and 21, and *beta-catenin* exon 3 showed no mutations. CGH analysis was performed as described previously [4] and revealed chromosomal gains at 1q, 6p, 6q24qter, 8q, 11q12q14, 11q23qter, 12q12q21, 12q24qter, 17q, 20q, losses at 5q14q23, 9p13pter, 13q21q21, and amplifications at 12q14q21, 15q24qter, 20q11q12 (Figure 3). CISH analysis verified an amplification of *MDM2*.

The diagnosis of the blastomatoid variant of pulmonary carcinosarcoma was established and the tumour was finally staged at pT3, pN0 (0/15), pMX, G3, R0, UICC stage 2b. The patient recovered well and was discharged 21 days after surgery. An optional adjuvant therapy was discussed with the patient, but he refused. Currently the patient is doing well and there is no evidence of tumour relapse 30 months after the resection.

The differential diagnoses of biphasic pleuropulmonary tumours in adults include glandular malignant peripheral nerve sheath tumour (MPNST), synovial sarcoma, and malignant mesothelioma [2,3,5]. When only small biopsy specimen are available and both components are not represented, carcinosarcoma may also be misinterpreted as an either entirely epithelial or mesenchymal neoplasm [2]. In glandular MPNST, rhabdomyosarcomatous elements may be present, but the tumour usually displays intestinal type-epithelium with goblet cells. Furthermore, the sarcomatoid part of MPNST expresses S100 protein and vimentin, and the tumour is associated with neurofibromatosis type 1 [6]. Synovial sarcoma was ruled out because the characteristic *SYT-SSX* fusion gene was not detected [7]. Malignant mesothelioma was also ruled out by negative staining for mesothelial markers (i. e. calretinin, D2-40).

Furthermore, pulmonary blastoma should be considered in the differential diagnosis if the epithelial component consists of adenocarcinoma [2]. As reported in the literature, biphasic pulmonary blastoma and carcinosarcoma, in particular the blastomatoid variant of the latter, may share common features, making a differentiation between both entities difficult [1,2,8]. Table 1 summarizes the clinicopathologic characteristics of pulmonary carcinosarcoma, pulmonary blastoma, and the present case. The blastomatoid variant of carcinosarcoma is not yet recognized as a distinct entity by the WHO classification of tumours [3]. In contrast to conventional pulmonary carcinosarcoma, which contains squamous cell carcinoma, adenocarcinoma, adenosquamous carcinoma, or large cell carcinoma as epithelial component, the blastomatoid variant of carcinosarcoma comprises high-grade adenocarcinoma of the fetal lung type/clear cell adenocarcinoma with fetal lung features [1]. This is a typical feature of pulmonary blastoma and therefore led to the designation as “blastomatoid pulmonary carcinosarcoma” [1].

**Table 1:** The clinicopathologic and molecular genetic characteristics of pulmonary blastoma, pulmonary carcinosarcoma, and the present case

**Diagnosis**	**Age**	**Sex**	**Incidence**	**Smoking**	**Location**	**Prognosis**	**Morphology**		**Immunohistochemical staining**		**Cytgenetic aberrations**	**Gene mutations**
							**Epithelial elements**	**Mesenchymal elements**	**Epithelial elements**	**Mesenchymal elements**		
Biphasic pulmonary blastoma [1,2,5-9]	35-52	M:F = 2:1	0.25-0.5% of pulmonary neoplasms	Yes	Upper lobe, central/endo-bronchial or peripheral	Poor (5-year survival rate 16%)	Low-grade adenocarcinoma of fetal lung type/well-differentiated fetal adenocarcinoma	Undifferentiated blastema, striated or smooth muscle, cartilage, bone, yolk sac-like areas, melanocytic differentiation, morules	EMA, pan-CK, CEA, TTF-1, CK7, nuclear/cytoplasmatic beta-catenin	Vimentin, desmin, SMA, myoglobin, S-100; morules: synaptophysin, chromogranin A, CD10	Trisomies 2 and 8, allelic imbalances at 14q24q32 and 17p11p13	*TP53*, *MDM2*, *beta-catenin* (no *EGFR* or *KRAS*)
Carcinosarcoma [1,2,4,5]	>50	M:F = 7:1	0.2-0.4% of pulmonary neoplasms	Yes	Upper lobe, central/endo-bronchial or peripheral	Poor (5-year survival rate 20-50%)	Squamous cell carcinoma, adenocarcinoma, adenosquamous carcinoma,large cell carcinoma	Spindle cells, fibrosarcoma, rhabdomyosarcoma, chondrosarcoma, osteosarcoma, blastema-like stroma	EMA, pan-CK, CK7, CAM5.2, CK5/6, p63, napsin, synaptophysin, chromogranin, CD56, membranous beta-catenin (TTF-1 negative)	Desmin, myogenin, myoD1, S-100	Gains: 1q, 3q, 5p, 8q, 12p; Losses: 3q, 5q, 17p	*TP53* (no *KRAS* or *beta-catenin*)
Present case: blastomatoid variant of carcinosarcoma	58	M	–	Yes	Right upper lobe	Alive, no relapse (22 months)	High-grade adenocarcinoma of fetal lung type	Spindle cells, rhabdomyosarcoma	EMA, pan-CK, CK7, CEA, MDM2, CDK4, focal p63, membranous beta-catenin (TTF-1 negative)	Vimentin, desmin, EGFR, CD56, myogenin, MDM2, CDK4	Gains: 1q, 6p, 6q24qter, 8q, 11q12q14, 11q23qter, 12q12q21, 12q24qter, 17q, 20q; Losses: 5q14q23, 9p13pter, 13q21q21; Amplifications: 12q14q21, 15q24qter, 20q11q12	*TP53, MDM2, CDK4* (no *EGFR*, *KRAS* or *beta-catenin*)

EMA: epithelial membrane antigen.

CK: cytokeratin.

CEA: carcinoembryonic antigen.

TTF-1: thyroid transcription factor 1.

CDK4: cyclin-dependent kinase 4.

In the case presented here, the gross appearance and localization of the tumour were not helpful in the differential diagnosis, since both pulmonary blastoma and carcinosarcoma are known to arise either central/peribronchial or in the periphery of the lung, forming a large bulk [2,8]. Pulmonary carcinosarcoma is reported to arise in elderly men between 50–80 years who are heavy smokers, as our patient [1,2,8,9], whereas the mean age of patients with pulmonary blastoma is around 35–50 years [2,10,11]. On microscopic examination, undifferentiated adenocarcinoma, intermixed rhabdomyosarcoma-like spindle cells, and extensive necroses may be observed in both entities [2,8]. It has been proposed to classify pulmonary blastoma into three groups: biphasic pulmonary blastoma, well-differentiated fetal adenocarcinoma, and pleuropulmonary blastoma, the latter arising only in children [10]. Biphasic pulmonary blastoma enters the differential diagnosis of carcinosarcoma as it combines malignant epithelial and mesenchymal components [10].

The epithelial component in the present case showed focal cellular atypia, mitotic figures, and branching glands composed of columnar cells with palisading elliptic nuclei with subnuclear vacuoles resembling high-grade fetal adenocarcinoma as described for carcinosarcoma [1]. In contrast, well-differentiated or low-grade adenocarcinoma would be a typical feature of pulmonary blastoma [1]. Morules, as described to occur in 43% of biphasic pulmonary blastomas, were not present [2,11]. The mesenchymal component displayed a rather immature, blastoma-like differentiation. Expression of TTF-1 occuring rather in the well differentiated fetal adenocarcinoma of pulmonary blastoma, was not observed in the present case, favouring the diagnosis of carcinosarcoma [9]. Furthermore, it has been demonstrated that low-grade adenocarcinoma of the fetal lung type/well-differentiated fetal adenocarcinoma constantly shows aberrant nuclear or cytoplasmic localization of beta-catenin, whereas high-grade adenocarcinoma of the fetal lung type/clear cell adenocarcinoma with fetal lung features shows the same membranous localization of beta-catenin as conventional pulmonary adenocarcinomas, which could also be observed in the present case [1].

Previous immunohistochemical and molecular analyses in pulmonary carcinosarcoma revealed mutations of *TP53*, but not of *KRAS* or *beta-catenin*[1,2]. To our knowledge, mutations of *EGFR*, *MDM2* or *CDK4* have not yet been investigated in this entity. Cytogenetic aberrations reported for this entity include allelic gains at 1q, 3q, 5p, 8q, 12p, and losses at 3q, 5q, 17p [2]. Pulmonary blastoma on the other hand is characterized by *TP53*, *MDM2*, and *beta-catenin* mutations, whereas *EGFR* and *KRAS* mutations are usually not detected [1,10,12,13]. Furthermore, trisomies 2 and 8, and allelic imbalances at 14q24q32 and 17p11p13 are reported for this tumour entity [2,10,12].

Immunohistochemically, an expression of p53 was detected in the present case consistent with an underlying TP53 mutation, but no *EGFR*, *KRAS* or *beta-catenin* mutation were found. Additionally, we observed a high number of chromosomal aberrations by CGH including gains at 1q, 6p, 6q24qter, 8q, 11q12q14, 11q23qter, 12q12q21, 12q24qter, 17q, 20q, losses at 5q14q23, 9p13pter, 13q21q21, and amplifications at 12q14q21, 15q24qter, 20q11q12. Interestingly, the observed gains at 1q, 8q, and losses at 5q are among the aberrations described for pulmonary carcinosarcoma [2]. The present case, however, displayed several more imbalances that have not yet been described for pulmonary carcinosarcoma. The high number of chromosomal imbalances indicates a high degree of chromosomal instability and tumour progression in the blastomatoid variant of carcinosarcoma. Furthermore, the observed imbalances may be of help in the differential diagnosis, in particular if +1q, +8q, and -5q are detected. The developmental origin of both tumour components is unclear and an origin from two or more stem cells (multiclonal hypothesis) or an origin from a single totipotential stem cell that differentiates into separate epithelial and mesenchymal directions (monoclonal hypothesis) seems possible [14]. Previous analyses in pulmonary carcinosarcoma [15], biphasic pulmonary blastoma [12], and carcinosarcomas of other localizations [14] provide evidence that the epithelial and mesenchymal component of these biphasic tumours harbour a different morphology, but are monoclonal in origin.

The observed chromosomal changes may give insight into tumourigenesis and help to identify possible candidate genes. The amplicon 12q13q21 (including the observed 12q14q21) is also typically detected in different types of sarcoma, particularly in liposarcoma and osteosarcoma [16]. It harbors several genes, of which the amplifications or mutations of *MDM2* and *CDK4* were confirmed immunohistochemically and by CISH analysis in the present case and may play a role in tumourigenesis [10,16]. Over-expression of MDM2 has previously been observed in 83% of biphasic pulmonary blastomas, but it has so far not been studied in pulmonary carcinosarcomas [10]. Furthermore, the observed amplicon at 15q24qter has also been reported in small-cell lung cancers, while the amplicon detected here at 20q11q12 includes the *PTPN1* gene located at 20q12 which serves as a non-receptor tyrosine phosphatase involved in growth regulation [16]. It has been found to be over-expressed in 72% of breast carcinomas [16].

As reported, complete surgical resection is the treatment of choice in patients with resectable tumours [2]. Chemotherapy and radiation can be used in an adjuvant setting although specific regimens do not exist [2,3]. In the present case, the mesenchymal component slightly predominated and an adjuvant therapy according to guidelines for soft tissue sarcomas was discussed. However, since complete resection was achieved, lymph node or distant metastases were ruled out, and the patient refused, no adjuvant therapy was applied.

The present case illustrates the diagnostic difficulties in differentiating pulmonary carcinosarcoma from biphasic pulmonary blastoma, in particular if the carcinosarcoma contains fetal adenocarcinoma and a blastomatoid mesenchymal component. It also highlights the usefulness of additional molecular and genomic analyses. From our findings we conclude that similar to pulmonary blastoma pulmonary carcinosarcoma may harbour *MDM2* and lack *EGFR* mutations. Therefore, presence of *TP53*, *MDM2*, and lack of *KRAS* and *EGFR* mutations may not be helpful in the differential diagnosis of both entities. Only the presence or absence of *beta-catenin* mutations will serve as a useful diagnostic tool in certain cases. In the present case, the diagnosis was discussed with reference pathologists who favoured the diagnoses of either pulmonary blastoma or blastomatoid carcinosarcoma. Although high-grade fetal adenocarcinoma was not present in every tumour sample, the diagnosis of pulmonary carcinosarcoma was finally made based on the mutation analyses, particularly of *beta-catenin*.

## Conclusions

In conclusion, the presence of high-grade fetal adenocarcinoma without TTF-1 expression, lack of *beta-catenin* mutation, and detection of 1q, +8q, and -5q by CGH in a biphasic lung tumour in adult patients favours the diagnosis of pulmonary carcinosarcoma. Furthermore, mutations of *TP53*, *MDM2*, *CDK4*, and *PTPN1* may play a role in development and progression of this tumour entity.

## Competing interests

The authors declare that they have no competing interests.

## Authors’ contributions

IMS and SS performed the histopathological, immunohistochemical and genetic examinations and established the diagnosis. COS, TO, and JM examined, treated and observed the patient, including follow-up. IMS, COS, TO, SS, and JM participated in writing the manuscript. COS and JM provided the radiographic, and IMS the histological and CGH images. All authors read and approved of the final manuscript.

## Consent

Written informed consent was obtained from the patient for publication of this Case report and any accompanying images. A copy of the written consent is available for review by the Series Editor of this journal.

## Pre-publication history

The pre-publication history for this paper can be accessed here:

http://www.biomedcentral.com/1471-2407/12/424/prepub
